# Thyroid-Stimulating Hormone Immunoassay Interference Presenting as Treatment-Refractory Hypothyroidism: A Case Report

**DOI:** 10.7759/cureus.108937

**Published:** 2026-05-15

**Authors:** Hajar Al Ismaili, Maisa Al Kiyumi

**Affiliations:** 1 Family Medicine, Wadi Bani Rawaha Health Center, Samail, OMN; 2 Family Medicine and Public Health, Sultan Qaboos University Hospital, Muscat, OMN

**Keywords:** biotin, free thyroxine, immunoassay interference, resistant hypothyroidism, thyroid-stimulating hormone

## Abstract

Measurement of thyroid-stimulating hormone (TSH) is fundamental for the diagnosis and therapeutic monitoring of hypothyroidism. Elevated TSH levels, with or without abnormalities in free thyroxine, typically indicate thyroid dysfunction and inform levothyroxine dose adjustments. Although suboptimal TSH control is most commonly attributed to poor medication adherence or improper administration, immunoassay-based TSH measurements are susceptible to analytical interference from sources such as biotin supplementation, heterophile antibodies, and macro-TSH complexes. These interferences may yield misleading biochemical results, potentially resulting in misdiagnosis, unnecessary investigations, and inappropriate escalation of therapy. We describe a case of a 53-year-old woman with persistently elevated TSH despite progressive levothyroxine dose escalation to 2.5 μg/kg/day, initially considered treatment-refractory hypothyroidism. Further evaluation ultimately identified TSH assay interference. This case highlights the importance of considering analytical artifacts when clinical and biochemical findings are discordant.

## Introduction

Hypothyroidism is a common endocrine disorder, particularly among women and adults over the age of 60, typically diagnosed biochemically by elevated thyroid-stimulating hormone (TSH) levels in conjunction with reduced or normal free thyroxine (FT4) concentrations. In most patients, levothyroxine replacement at standard weight-based doses (approximately 1.5-1.8 μg/kg/day) effectively restores euthyroidism and normalizes TSH levels [1]. However, some patients continue to have elevated TSH despite apparently adequate or escalating levothyroxine therapy, a condition referred to as resistant or treatment-refractory hypothyroidism [2,3]. Treatment refractoriness in hypothyroidism is most commonly attributed to a specific and identifiable set of contributing factors. These include poor medication adherence, impaired gastrointestinal absorption of levothyroxine, and co-administration of interfering substances such as proton-pump inhibitors, calcium supplements, and iron preparations. Biotin supplementation may also contribute by interfering with streptavidin-based immunoassays, producing falsely abnormal thyroid hormone measurements. Additionally, laboratory assay interference from heterophilic antibodies, macro-TSH complexes, or anti-streptavidin antibodies may produce spuriously elevated TSH values, mimicking biochemical treatment failure without true thyroid dysfunction [3,4]. Early and systematic identification of these factors is critical, as failure to do so may result in unnecessary dose escalation and expose patients to the risks of iatrogenic thyrotoxicosis and its associated complications.

## Case presentation

A 53-year-old woman presented with complaints of persistent fatigue and menorrhagia of prolonged duration. She was first evaluated at our Health Center in 2022. Initial laboratory investigations revealed a low hemoglobin level of 8.8 g/dL (reference range: 11-14.5 g/dL), a low mean corpuscular volume of 54.2 fL (reference range: 78-95 fL), and a low mean corpuscular hemoglobin of 17 pg (reference range: 26-33 pg). Thyroid function testing showed a markedly elevated TSH level of 23 mIU/L (reference range: 0.27-4.5 mIU/L) with a normal free T4 level of 12.87 pmol/L (reference range: 12-22 pmol/L). She was managed as having hypothyroidism and started on 50 µg of thyroxine daily. As part of her evaluation, she was referred for gynecological consultation for menorrhagia at a tertiary hospital and underwent an endometrial biopsy, which was reported as normal. During this period, she was also referred to a physician for optimization of her hypothyroidism. Additional investigations revealed an elevated thyroid peroxidase antibody level at 29.7 IU/L (reference range: 0-5.9 IU/L), negative thyrotropin receptor antibodies, and a normal thyroid ultrasound. Throughout her treatment journey, her thyroxine dose was adjusted multiple times and eventually increased to 150 µg per day; however, her TSH levels remained elevated. After discharge from the gynecologist, the patient returned to our health center in 2023 for continuation of care. Her TSH level remained elevated at 17.7 mIU/L with a free T4 level of 33 pmol/L, necessitating a further dose increase to 175 µg/day (approximately 2.5 µg/kg/day). This dose substantially exceeds the standard weight-based replacement dose of 1.5-1.8 µg/kg/day recommended for most hypothyroid patients, thereby meeting the criteria for treatment-refractory hypothyroidism. Treatment-refractory hypothyroidism is defined as persistent biochemical hypothyroidism despite adequate and optimized levothyroxine therapy [2,3]. Medication adherence was assessed through direct patient interview and prescription refill records, both of which confirmed consistent and appropriate levothyroxine intake. The patient reported taking levothyroxine in a fasting state, at least 30-60 minutes before breakfast, in accordance with standard administration guidelines. A thorough medication and supplement review was conducted, specifically excluding the use of calcium supplements, iron preparations, proton-pump inhibitors, and biotin, all of which are known to interfere with levothyroxine absorption or assay accuracy. Furthermore, the patient denied any gastrointestinal symptoms suggestive of malabsorption, such as chronic diarrhea, bloating, or steatorrhea, and had no prior history of gastrointestinal pathology that could impair thyroid hormone absorption.

At this stage, the patient was referred to the National Diabetic and Endocrine Center for further evaluation and management, with possible pituitary magnetic resonance imaging planned. Surprisingly, repeat testing demonstrated a suppressed TSH level (<0.001 mIU/L), suggesting assay interference. The main interfering antibodies addressed in Roche platforms include heterophile antibodies. These are weak, nonspecific human antibodies that can bind assay antibodies and create false signals. Roche assays contain blocking agents (nonimmune immunoglobulins and proprietary blockers) designed to reduce this interference.

Her thyroxine dose was reduced to 50 µg. She was advised to follow up at the health center, where it was recommended that she have her tests performed at the National Diabetic and Endocrine Center’s laboratory; however, no clear referral was provided. She was seen again in August 2024, and the TSH level tested at the regional hospital laboratory was reported as 12.5 mIU/L, with a T4 level of 14.98 pmol/L. Accordingly, her thyroxine dose was again titrated upward, eventually reaching 175 µg. The patient was subsequently reviewed at our clinic in June 2025, as her TSH level remained elevated at 21 mIU/L, with a high T4 level of 28.9 pmol/L. Her results and summary file were carefully reassessed. On this occasion, her sample was again sent to the National Diabetes and Endocrine Center, and results revealed a suppressed TSH level (<0.008 mIU/L) with a T4 level of 28.4 pmol/L. Her thyroxine dose was then gradually reduced, with frequent four-weekly TSH monitoring, until target levels were achieved. Her most recent TSH level was 1.21 mIU/L, and she was maintained on a dose of 50 µg of thyroxine. She has continued regular follow-up and remains well controlled on this dose. Importantly, thyroid function testing in our patient was performed using the Roche immunoassay platform at the National Diabetes and Endocrine Center, whereas previous tests at the local health center had been analyzed using the Siemens platform. This inter-platform comparison underscores the importance of considering and mitigating potential assay interference, including the effect of interfering antibodies. Refer to Table 1 for a summary of the patient’s thyroid function test results.

**Table 1:** Summary of the patient’s thyroid function test results (TSH and free thyroxine measurements) TSH, thyroid-stimulating hormone

Date	TSH (mIU/mL)	Free T4 (pmol/L)	Platform used
31/8/2022	23.02	13.6	Siemens platform
24/8/2024	12.5	14	Siemens platform
16/10/2024	13.8	16.1	Siemens platform
8/12/2024	11.3	23.4	Siemens platform
9/2/2025	14.2	26.7	Siemens platform
20/4/2025	18.9	23.12	Siemens platform
24/6/2025	21.0	28.9	Siemens platform
30/6/2025	<0.008	28.4	Roche immunoassay
3/8/2025	<0.008	19	Roche immunoassay
8/9/2025	0.35	13	Roche immunoassay
20/10/2025	1.21	-	Roche immunoassay

## Discussion

Refractory hypothyroidism is increasingly recognized in clinical practice and is generally defined by persistently elevated TSH levels despite progressive escalation of levothyroxine therapy, regardless of the patient's symptoms. Unchecked escalation to supratherapeutic doses carries substantial risks, including iatrogenic thyrotoxicosis, arrhythmias, and heart failure, and therefore should prompt careful systematic reassessment rather than empirical dose intensification. Current evidence supports a structured approach in which medication adherence is verified first, followed by evaluation for impaired absorption, drug-food interactions, increased metabolic demands, or analytical assay interference when biochemical targets remain unmet [1,2].

Treatment-refractory hypothyroidism most commonly results from remediable clinical or analytical factors rather than true failure of hormone replacement. The most common cause is poor adherence to levothyroxine therapy, followed by conditions that increase hormone requirements, such as pregnancy or substantial weight gain, or enhance levothyroxine clearance through enzyme-inducing drugs, including phenobarbital, phenytoin, carbamazepine, and rifampicin. Impaired absorption is another key contributor and may result from dietary influences, including coffee, soy products, high-fiber foods, or certain herbal supplements; from interacting medications such as proton-pump inhibitors, sucralfate, bile-acid sequestrants, iron, or calcium; or from gastrointestinal disorders, including autoimmune atrophic gastritis, Helicobacter pylori infection, small-intestinal bacterial overgrowth, celiac disease, lactose intolerance, or short-bowel syndrome. Laboratory artifacts should also be considered, including TSH assay interference from heterophilic antibodies, macro-TSH, biotin exposure, anti-streptavidin or anti-ruthenium antibodies, and thyroid hormone autoantibodies, which can produce misleading biochemical results that mimic treatment resistance [1].

The diagnostic challenge of apparent biochemical resistance in thyroid disease is illustrated by cases of immunoassay interference. Ansar et al. described a 57-year-old woman with primary hypothyroidism who had persistently elevated TSH levels that did not match her clinical status or levothyroxine adjustments; extensive evaluation ultimately identified heterophile antibody interference as the cause of falsely raised TSH values [3]. Similarly, de Silva et al. reported a five-year-old girl evaluated for short stature who had persistently elevated TSH levels with normal FT4 levels despite being clinically euthyroid and receiving escalating levothyroxine doses for a year. Suspecting assay interference, repeat testing using an alternative platform and after removal of heterophile antibodies revealed normal TSH levels, confirming the interference as the source of abnormal results [5].

Macro-TSH, a high-molecular-weight complex formed by TSH binding to circulating immunoglobulins, represents another significant cause of persistently elevated TSH that can mimic subclinical hypothyroidism or apparent resistance to levothyroxine therapy. Few case reports highlight this diagnostic pitfall. D'Arcy et al. described a patient whose TSH remained elevated despite escalating levothyroxine doses until macro-TSH was identified, preventing unnecessary over-treatment [6]. Similarly, Yalcin et al. reported markedly elevated TSH despite increasing thyroxine doses, with polyethylene glycol precipitation confirming macro-TSH as the source of persistently elevated TSH levels [7]. More recent literature continues to emphasize this entity, including a case by Jin et al. demonstrating falsely elevated TSH due to macro-TSH and highlighting the need to reconsider laboratory interference in apparently refractory cases, and a clinical discussion by Chiardi et al. highlighting that recognition of macro-TSH is essential to avoid misdiagnosis and inappropriate levothyroxine dose escalation [8,9].

Platform-specific immunoassay interference, particularly from antiruthenium antibodies, is another important but under-recognized cause of misleading thyroid function results that can simulate refractory hypothyroidism and lead to inappropriate levothyroxine dose escalation. Buijs et al. documented several cases in which anti-ruthenium antibodies interfered with electrochemiluminescence immunoassays, producing falsely abnormal TSH and thyroid hormone measurements that normalized when alternative testing methods were used [10]. Subsequent reports and reviews have similarly described patients with primary hypothyroidism in whom spuriously elevated TSH due to anti-ruthenium antibody interference was identified only after assay comparison, underscoring the need to consider analytical interference whenever biochemical results do not align with clinical findings or treatment response [4].

## Conclusions

Although uncommon, TSH assay interference represents an important and often overlooked potential explanation for apparent treatment-refractory hypothyroidism. In this case, the biochemical findings were highly suggestive of and consistent with TSH assay interference, particularly given the progressive levothyroxine dose escalation that resulted in supratherapeutic FT4 concentrations without the expected degree of TSH suppression. While formal confirmatory interference studies were not performed, the overall clinical and biochemical picture did not support true pharmacological refractoriness, and the possibility of assay interference was considered the most plausible explanation for the observed discordance. These findings highlight the importance of maintaining a high index of suspicion for assay interference when thyroid biochemistry fails to align with the clinical presentation. In such circumstances, repeat measurement on an alternative immunoassay platform or the application of established interference mitigation methods, such as polyethylene glycol precipitation or heterophilic antibody blocking, should be considered as the next diagnostic step, with the aim of preventing unnecessary investigations, inappropriate dose escalation, and potential iatrogenic harm.
